# Cross-cultural adaptation and psychometric validation of the Kannada version of the Extended Effort Assessment Scale (EEAS-K) in adults with hearing loss

**DOI:** 10.1371/journal.pone.0358439

**Published:** 2026-09-23

**Authors:** Bhavana Rhythm, Kanaka Ganapathy, Archana Gundmi, Hari Prakash Palaniswamy

**Affiliations:** Department of Speech and Hearing, Manipal College of Health Professions, Manipal Academy of Higher Education, Manipal, India; Gallaudet University, UNITED STATES OF AMERICA

## Abstract

**Introduction:**

The Extended Effort Assessment Scale (EEAS) is a recently developed questionnaire for measuring listening effort, but a validated version for Kannada speakers is lacking. This study aimed to translate, culturally adapt, and psychometrically validate the EEAS for the Kannada-speaking adults with hearing loss.

**Method:**

The EEAS was translated into Kannada (EEAS-K) following rigorous forward and back translation, expert committee review and cognitive debriefing. The final EEAS-K was then field-tested with 100 native Kannada-speaking adults with hearing loss (predominantly non-hearing aid users, 92%). Psychometric validation included assessing construct validity via exploratory factor analysis, Rasch analysis using item response theory (IRT), internal consistency (Cronbach’s alpha), convergent validity via a Visual Analogue Scale (VAS), test-retest reliability with an Intraclass correlation coefficient (ICC) and Differential Item Functioning (DIF).

**Results:**

All 10 EEAS-K items demonstrated acceptable content validity (CVR **=** 1.00). EFA, guided by eigenvalues and the scree plot, provided initial support for a unidimensional structure, with a single factor accounting for 32.7% of the variance. Rasch analysis supported essential unidimensionality (first residual eigenvalue = 1.90). Nine of 10 items showed acceptable infit MNSQ values (0.64–1.34); Q8 (telephone) showed elevated infit (1.92) and outfit (1.83) values, indicating misfit, which may reflect the unique and multidimensional cognitive demands of technology-mediated communication in this population. Person-item targeting remained within acceptable clinical limits, though items were slightly easier for the average person’s ability (person mean = 0.49 logits). The EEAS-K demonstrated excellent internal consistency (α = 0.886), moderate to strong convergent validity with the VAS (r = 0.611), and excellent test-reliability (ICC = 0.91). DIF analysis identified significant gender-based DIF for Q7 and age-based DIF for Q7 and Q8, suggesting raw scores for these specific items should be interpreted with caution across these demographics. No significant DIF was found for hearing-loss-severity.

**Conclusion:**

The EEAS-K is a reliable and valid tool for assessing listening effort in Kannada-speaking adults with hearing loss. The Wright map demonstrates adequate person-item targeting, and the dual-method psychometric approach provides satisfactory measurement evidence supporting clinical and research use.

## Introduction

Listening Effort is defined as “the deliberate allocation of mental resources to overcome obstacles in goal pursuit when carrying out a task, with listening effort applying more specifically when tasks involve listening.” [1]. This complex, multifaceted concept has attracted considerable attention in hearing science research, as it is particularly relevant in contexts that require focused auditory attention. For individuals with hearing loss, the burden of sustained listening effort is a critical concern, as it can lead to severe cognitive fatigue, stress, and social withdrawal [2]. Consequently, listening effort is increasingly recognised as an important clinical outcome beyond pure-tone audiometry. While the audiogram effectively measures peripheral hearing sensitivity, it fails to capture the top-down cognitive resources and real-world communication struggles patients experience when trying to decode degraded speech signals [3]. Despite its clinical significance, there is a notable shortage of validated questionnaires that specifically measure perceived listening effort in everyday, real-world listening environments, particularly in Indian languages.

Self-reported questionnaires offer a practical and efficient means of assessing perceived listening effort from the individual’s perspective. Commonly used subjective assessment tools include Visual Analogue Scales (VAS) and the National Aeronautics and Space Administration Task Load Index (NASA-TLX) [4,5]. While generic measures like the NASA-TLX capture overall cognitive load, they may not adequately address the specific challenges encountered in listening situations [6]. Condition-specific questionnaires offer distinct advantages over these generic measures because they are tailored to capture the unique, multidimensional acoustic and communicative challenges specifically experienced by individuals with hearing loss.

In recent years, demand for refined assessment tools has increased, leading to the development of specialised questionnaires focused on listening effort. Instruments such as the “listening effort” sub-sections of the Speech, Spatial, and Qualities of Hearing Scale (SSQ), the Effort Assessment Scale (EAS), and the Extended Effort Assessment Scale (EEAS) offer valuable insights into this phenomenon, particularly for hearing aid users [7–9].

The Effort Assessment Scale (EAS; Alhanbali et al., 2023) is the first questionnaire specifically designed to measure listening effort in everyday listening situations. The EAS has been translated and psychometrically validated in several languages, including Danish and Arabic, with these versions demonstrating good reliability and validity [10,11]. However, as noted by the developers of the extended scale, the EAS has a limitation: in three of its six items, the specific listening environment of whether noisy or quiet has not been clearly defined, leaving interpretation to the individual [9]. This lack of clarity may influence response consistency, particularly for individuals with hearing impairments, who may face varying levels of difficulty depending on the environmental conditions observed during pilot testing of EAS, which revealed that respondents answered differently depending on whether they interpreted the situation as occurring in a quiet or noisy environment.

To address these limitations, the Extended Effort Assessment Scale (EEAS) was developed by Ferschneider and Moulin (2023). The EEAS builds upon the original EAS by assessing listening effort separately in both quiet and noisy environments. It consists of 10 items designed to quantify the subjective listening effort of individuals with hearing loss, including hearing aid users. This provides a more nuanced and comprehensive measure of the challenges faced in everyday listening situations. In its original English validation study, the EEAS demonstrated excellent internal consistency and a clear two-factor structure representing effort in quiet and effort in noise, proving to be a robust tool for clinical use. However, for effective use among non-English-speaking populations, rigorous cross-cultural adaptation, linguistic validation and psychometric validation are essential [12].

The current study aimed to translate, culturally adapt, and psychometrically validate the EEAS for Kannada-speaking adults with hearing loss.

## Methods

### Ethical clearance

The study protocol received approval from the Institutional Ethical Committee of Kasturba Hospital (IEC 445/2024), and the study was registered with the Clinical Trials Registry of India (CTRI/2025/03/082719). Permission was obtained from the original authors before the translation process commenced. These guidelines are based on the National Ethical Guidelines for Biomedical Research on Human Participants by the Indian Council of Medical Research (ICMR, 2017), which adhere to the principles of the Declaration of Helsinki. Participants were recruited for this study between May 2025 and October 2025. Written informed consent was obtained from all participants before their inclusion in the study.

### Overview

**Fig 1:**
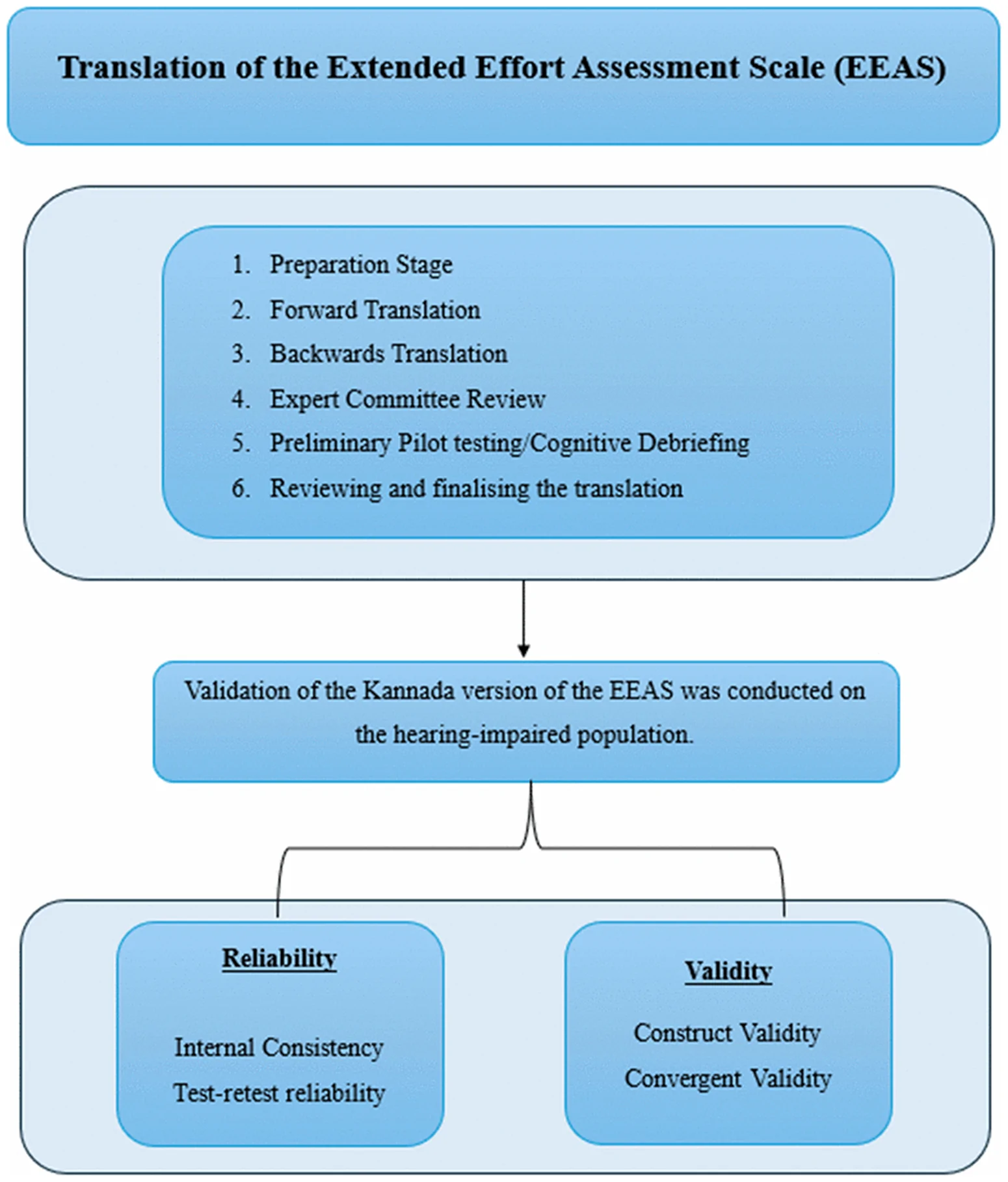
Schematic representation of the translation and validation process for the Kannada version of the EEAS.

The study was carried out in two phases. Phase I involved adaptation of the EEAS, and Phase II focused on field testing and validation of the Kannada version of the EEAS (EEAS-K). The adaptation process followed the good practice guidelines for hearing-related questionnaires by Hall et al. (2018), involving forward translation, reconciliation, back translation, expert committee review, and cognitive debriefing is illustrated in Fig 1.

### Phase 1: Translation of the Extended Effort Assessment Scale (EEAS)

The translation and cultural adaptation of the EEAS followed the guidelines for cross-cultural adaptation of health-related questionnaires [13]. To comply with copyright, permission was sought from the original developers to proceed with the translation.

**Forward Translation Process.** Two bilingual translators, proficient in both English and Kannada, independently performed the forward translation. They were instructed to ensure that the translations maintained equivalent meaning, accessibility, and terminology based on the concept definitions provided by the original test developer. During this stage, several translation choices were made to ensure cultural and linguistic appropriateness. For example, the word “restaurant” was translated to “hotel”, as this is the more common term for an eating establishment in everyday Kannada. Similarly, for the word “telephone”, the formal Kannada word was used, with the more familiar English word retained in brackets for clarity. For modern concepts without direct Kannada equivalents, such as “train station,” “airport”, and “stadium”, direct transliterations were used. This process yielded two distinct translations of the scale (F1 and F2). Discrepancies between the two forward translators were resolved through consensus, with a third independent reviewer (a senior audiologist with comparable linguistic proficiency) adjudicating any unresolved differences, in accordance with Hall et al. (2018). All decisions and discrepancies recorded in a reconciliation report.

**Backward Translation Process.** The reconciled forward translation was then given to a new bilingual translator for back-translation into English. This translator, a professional linguist who had not seen the original English EEAS, was proficient in both English and Kannada and was tasked with ensuring the translated items accurately reflected the intent of the Kannada version. This step is crucial for identifying any misinterpretations or loss of meaning that may have occurred during the forward translation. A single professional back-translator was utilized, which meets the recommended minimum standard for the cross-cultural adaptation of hearing-related questionnaires [13].

**Expert Committee Review.** The expert committee, comprising five senior audiologists with at least 5 years of clinical experience and native proficiency in Kannada, was then convened. The committee was provided with the original English EEAS, the reconciled forward translation, and the back-translation. Their task was to review all three documents to assess for any discrepancies and to validate the conceptual and cultural equivalence of the translated items. The five expert audiologists independently rated each item without prior group discussion. To ensure the items’ content validity, the items were assessed using the Content Validity Ratio (CVR) and the Content Validity Index (CVI). The CVR was computed using the formula CVR = [ne – (N/2)] / (N/2), where ne is the number of expert panelists rating an item as “essential” and N is 5. An I-CVI **≥** 0.78 was used as the threshold for acceptable item-level content validity. The committee formally approved the translation choices made in the forward translation stage, including the cultural adaptation of “hotel” and the retention of the English word “telephone,” confirming that these decisions enhanced the clarity and relevance of the questionnaire for the target population.

**Cognitive debriefing.** In this stage, the EEAS-K questionnaire was administered to Kannada-speaking participants with hearing loss.A sample of five participants was selected for cognitive debriefing, aligning with the recommended group size of five to eight individuals for pre-testing hearing-related questionnaires [13,14]. They were instructed to provide a descriptive explanation of the questionnaire’s understandability and alternatives to difficult-to-comprehend words. Item modifications were considered if 2 out of 5 individuals found the item unclear. This threshold helped us distinguish between a real issue with the translation and a single participant’s personal difficulty. A few Kannada words, such as “underground station” and “stadium,” were a little unfamiliar, as they are not part of their daily usage. However, they could relate and understand the meaning of those words. Hence, those Kannada words were retained in the questionnaire. Another significant observation during this phase was that participants tended to form a response to the core question before reaching the environmental context (e.g., ‘in a quiet environment’), which was placed at the end of the sentence. This could lead to confusion or require participants to re-evaluate their initial judgment. This method provided an appropriate linguistic validation of the scale.

**Reviewing and Finalising the Translation.** Based on the crucial feedback from the cognitive debriefing, a final structural modification was made to all relevant items; the environmental context was moved to the beginning of the sentence (e.g., ‘In a quiet environment, do you have to put in a lot of effort...? ‘). This change ensures that the participant is primed with the listening context before considering the effort required. While this structural modification altered the syntax to align with the Kannada grammatical rules, the semantic and conceptual equivalence of the listening scenarios was fully preserved [13]. The finalised Kannada version was then proofread for typographical and formatting errors by a native Kannada-speaking reviewer. After this final revision, the 10-item EEAS-K was drafted and prepared for administration during the Phase II validation.

### Phase II: Validation of EEAS-K using field testing

The current study employed a cross-sectional design. Participants were recruited using consecutive sampling from the Department of Speech and Hearing at Kasturba Hospital in Manipal, India, with complaints of reduced hearing sensitivity were approached. The study procedures were explained to all potential participants, and those who provided written informed consent were included.

### Participants

A total of 100 participants were recruited for the study’s validation phase, based on the recommendation of 10 participants per questionnaire item [13]. The sample included native Kannada speakers aged 18–59 years. Audiological evaluations were conducted using a Madsen Astera audiometer with supra-aural TDH-39 headphones, calibrated in accordance with ANSI standards. Pure-tone average (PTA) was calculated using thresholds at 500, 1000 and 2000 Hz. Hearing loss severity was classified according to Goodman’s classification, ranging from minimal to profound. The primary inclusion criterion was having subjective complaints of hearing difficulties. Therefore, while the sample was predominantly bilateral (n = 98), a small number of participants (n = 2) with unilateral hearing loss who reported significant listening effort were included. Participants with purely conductive hearing loss were excluded. Educational eligibility required completion of more than the 8th standard of education to ensure adequate literacy and reading comprehension for the self-administered questionnaire. Participants with self-reported neurological conditions (such as Parkinson’s disease, dementia, or a history of stroke) or psychological impairments (including anxiety and depression) were excluded.

### Procedure

Once consent was obtained, demographic details, including age, gender, and duration of hearing loss, were recorded. The researcher then explained the study to each participant. The translated EEAS-K was administered to all participants, requiring approximately 10 minutes to complete. Participants who used hearing aids completed the questionnaire based on the typical daily aided listening experience. To assess convergent validity, a single VAS question was included along with the demographic form. Participants were asked to rate the impact of their listening-related effort on their daily lives using a 10-point scale, where 0 indicated “no effort,” and 10 indicated “high effort.” Both questionnaires were self-administered, but interview assistance was provided to participants who required it.

### Scales and subscale score calculation

The EEAS subscale structure was based on the original English validation study to maintain consistency with the established psychometric framework.‘Not Applicable’ (NA) responses were scored as 0, as a scenario that does not apply requires no listening effort. Different EAS scores were calculated as follows:

- EEAS*quiet* (average of Q1, Q3, and Q8) representing listening effort in quiet environments.
- EEAS*noise3* (average of Q2, Q4, and Q9) representing listening effort in noisy environments for the same types of listening situations as the quiet subscale.
- EEAS*noisy-quiet* represents the difference score between noisy and quiet conditions.
- EEAS*noise* (average of Q2, Q4, Q5, Q6, Q7, Q9, and Q10) representing all items related to noisy listening environments.
- EEAS total score representing the average of all 10 items.

The possible score range is 10–50 for the Total EEAS, 4–20 for the EEAS-quiet subscale, and 4–20 for the EEAS-noise subscale. Across the total scale and all subscales, higher scores consistently indicate a greater degree of perceived listening effort.

### Statistical analysis

All classical test theory analyses were performed using Jamovi (Version 2.3.21.0). IRT/Rasch analysis was performed using a Python-based script utilising the statsmodels and scipy libraries.

To evaluate validity, Content Validity Ratio **(**CVR) was computed for each item. I-CVI **≥** 0.78 was used as the threshold for acceptable item-level content validity. For Exploratory Factor Analysis (EFA), sampling adequacy was assessed using the Kaiser-Meyer-Olkin (KMO) statistic and Bartlett’s test of sphericity. EFA was conducted using maximum likelihood extraction with an oblimin rotation, extracting 1–4 factor solutions. Factor retention was determined based on eigenvalues greater than 1.0 and visual inspection of the scree plot, supplemented by the Bayesian Information Criterion (BIC). The root-mean-square error of approximation (RMSEA), the comparative fit index (CFI), the Tucker-Lewis Index (TLI), and the standardized root-mean-square residual (SRMR) are also reported. Convergent validity was assessed using Pearson’s correlation coefficients to examine the association between the EEAS total score and its subscales and the VAS ratings, and better-ear PTA.A single-item VAS was utilized because no other validated, condition-specific listening effort questionnaires currently exist in the Kannada language for comparison. Strong positive correlations were expected to support convergent validity.

To assess reliability, internal consistency was assessed by calculating both item-to-total correlation and Cronbach’s α for the total scale and each subscale. Cronbach’s alpha was calculated to facilitate direct comparisons with the original EEAS validation study and existing literature based on Classical Test Theory. A higher item-rest correlation (> 0.30) was considered indicative of greater consistency with the rest of the scale. To evaluate the scale’s reliability over time, test-retest reliability was assessed in a subset of 20 participants who completed the EEAS-K a second time, two to three weeks after their initial assessment. Participants did not undergo any new hearing interventions, hearing aid adjustments, or changes in clinical management between the two testing sessions. Identical administration conditions (e.g., a quiet clinical room, paper-and-pencil format) were strictly maintained during the second assessment to minimise environmental measurement variability. Test-retest reliability was calculated using a two-way intraclass correlation coefficient (ICC) model with absolute agreement for single measures. This model was selected because the same rater administered the test to all participants across both time points, and absolute agreement is necessary to ensure scores remain stable over time. ICC values > 0.70 were considered acceptable [15].

To evaluate item-level measurement quality, Rasch analysis was performed using a Poisson regression approach to Joint Maximum Likelihood Estimation (JMLE) to calculate item difficulties and fit statistics. The original 0–10 response categories were collapsed into a 4-category scale (0–2 = 0; 3–5 = 1; 6–8 = 2; 9–10 = 3) before analysis to resolve disordered thresholds and ensure monotonic advancement of step calibrations. This decision was guided by Linacre’s (2002) criteria for optimising rating scale category functioning, ensuring each category had a sufficient frequency of observations [16].

Item difficulty was estimated on the logit scale. Item fit was evaluated using unstandardized Infit and Outfit Mean Squared Error (MNSQ) values, as standardised Z-values (ZSTD) are highly sensitive to sample size and MNSQ provides a more robust indicator of practical misfit for samples of this size (Linacre,2002). Values between 0.70 and 1.30 are considered productive for measurement; values > 1.50 indicate misfit (unexpected variation); values < 0.50 indicate overfit (too predictable, reducing item utility) [16]. Person-item targeting was assessed via a Wright Map (Person-Item Map), which plots the distribution of person ability and item difficulty on the same logit scale.

Differential item functioning (DIF) was assessed across three subgroup variables: Gender (male vs female), Age (younger ≤ 53 years vs older > 53 years, split at median), and Degree of hearing loss (Better PTA ≤ 30 dB vs Poorer PTA > 30 dB, split at median). DIF was evaluated using an Ordinary Least Squares (OLS) regression-based method, testing the main effect of group membership on item scores while controlling for overall ability (rest-score). The statistical significance was set at p < 0.05, and the magnitude of DIF was evaluated based on the resulting regression coefficients.

## Results

### Participants characteristics

The study included 100 participants. The overall rate of “Not Applicable” (NA) responses was low (1.6% of all possible responses), recorded when participants indicated that listening scenarios did not apply to their daily experiences. Formal evaluation of raw scores revealed no significant floor or ceiling effects, as less than 15% of the sample achieved the absolute minimum or maximum possible scores. Table 1 presents the demographic and clinical characteristics of the study sample.

**Table 1:** Participant Characteristics.

Characteristics	Value (n = 100)	
**Age, years**	48.6 ± 11.1 (18-59)	
**Gender, n (%)**		
**Male**	60(60%)	
**Female**	40(40%)	
**Type of hearing loss, n (%)**		
**Sensorineural/Mixed**	100 (100%)	
**Conductive**	0 (0%)	
**Hearing aid use, n (%)**		
**No**	92 (92%)	
**Yes**	8 (8%)	
**Hearing aid usage duration**	1 month to 4 years	
**Right ear PTA, dB HL**	44.5 ± 22.5 (12-112)	
**Left ear PTA, dB HL**	43.3 ± 19.4 (12-89)	
**Better ear PTA, dB HL**	34.6 ± 17.2 (12-89)	
**Hearing asymmetry, dB**	18.6 ± 17.7 (0-67)	
**Laterality of hearing loss, n (%)**		
**Bilateral**	98 (98%)	
**Unilateral**	2 (2%)	
**Degree of Hearing Loss, n**	**Right Ear**	**Left Ear**
**Minimal (≤25 dB HL)**	26	24
**Mild (26–40 dB HL)**	25	29
**Moderate (41–55 dB HL)**	19	17
**Moderately Severe (56–70 dB HL)**	16	18
**Severe (71–90 dB HL)**	9	12
**Profound (≥ 91 dB HL)**	5	0
**EEAS total score**	4.4 ± 1.9 (1-8.9)	
**VAS score**	4.7 ± 2.2 (0-10)	

**Note:** Data are presented as mean ± standard deviation (range) for continuous variables and n (%) for categorical variables. Hearing loss severity is based on Goodman’s classification. **VAS** = Visual Analogue Scale

The participants’ mean age was 48.6 years (SD ± 11.1, range: 18–59 years), with a gender distribution of 60 males (60%) and 40 females (40%). Only 8% reported using a hearing aid. Audiometric assessment revealed varying degrees and configurations (predominantly sloping and flat) of hearing loss across the sample. The mean better ear PTA was 34.6 dBHL (SD = 17.2), and the ear asymmetry showed considerable variability (M = 18.6 dB, SD ± 17.7).

### Content validity

Both the Item-Level Content Validity Index (I-CVI) for all individual items and the overall Scale-Level Content Validity Index (S-CVI) were 1.00, indicating excellent content validity.

### Construct validity

**Fig 2:**
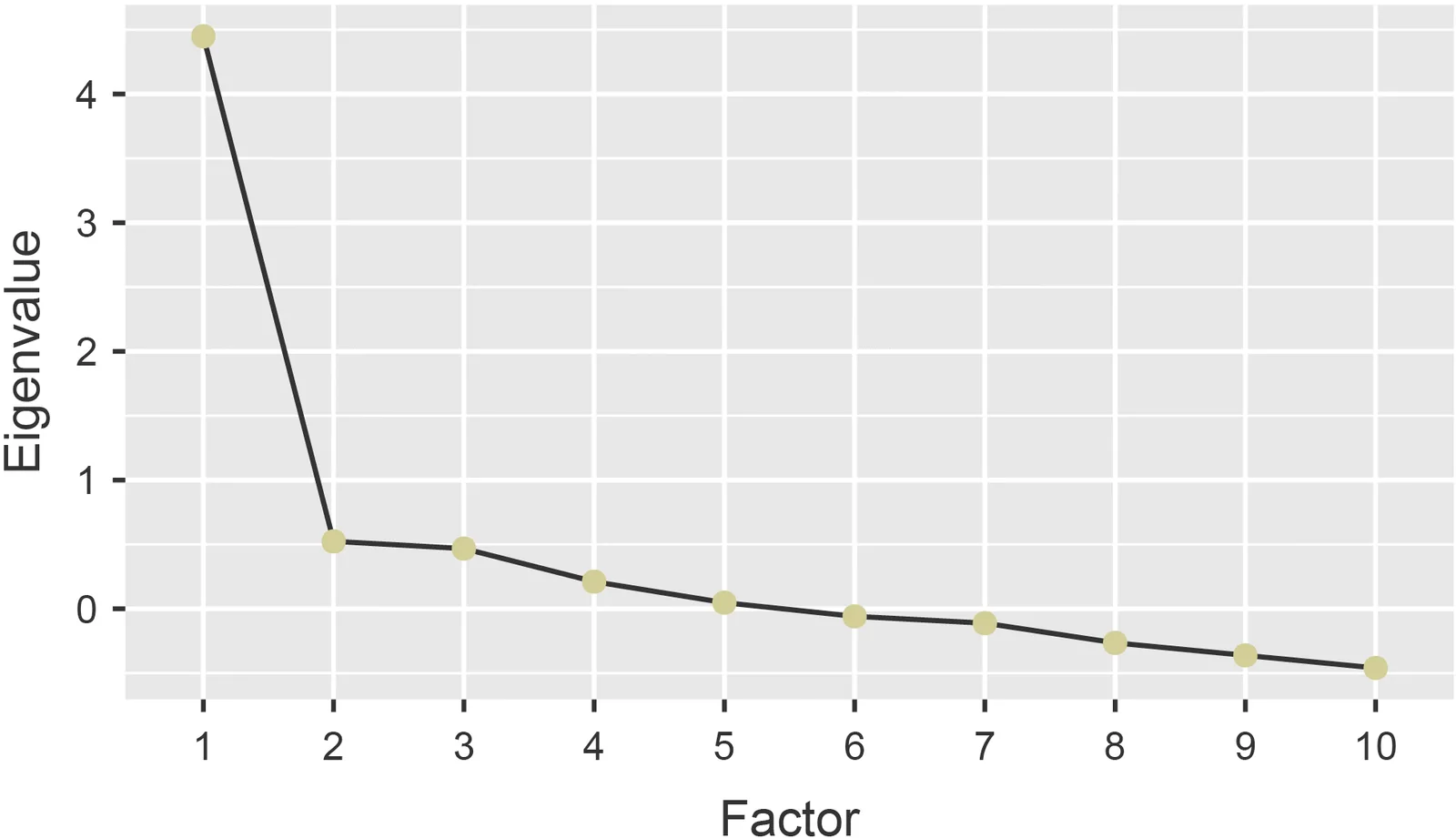
Scree Plot.

Sampling adequacy was assessed using the Kaiser-Meyer-Olkin (KMO) measure, which yielded a value of 0.740 with individual item KMO values ranged from 0.666 to 0.827, all exceeding the recommended threshold of 0.60. Bartlett’s test of sphericity was highly significant (χ² = 537, df = 45, p < .001), confirming that the data were eligible for factor analysis. Exploratory factor analysis was performed using maximum likelihood extraction with oblimin rotation, extracting 1, 2, 3, and 4 factor solutions. Model selection was guided by eigenvalues, as presented in the scree plot (Fig 2), and model fit indices (Table 2). The scree plot showed a steep drop after the first factor (eigenvalue = 4.44), with all subsequent eigenvalues falling below 1.0 (eigenvalue = 0.52), strongly indicating a unidimensional structure. The one-factor solution was therefore retained as the most parsimonious and psychometrically defensible model.

**Table 2:** Factor analysis results based on the EEAS questionnaire.

Fit indices	1. Factor	2. Factor	3. Factor	4. Factor
RMSEA	0.203[0.176,0.235]	0.195[0.163,0.232]	0.180[0.141,0.224]	0.179[0.128,0.235]
TLI	0.617	0.645	0.694	0.698
CFI	0.835	0.888	0.933	0.960
SRMR	0.104	0.200	0.240	0.206
BIC	19.1	5.49	−6.14	−4.44

**Note:** RMSEA: root-mean-square error of approximation; TLI: Tucker-Lewis index; CFI: comparative fit index; SRMR: Standardised root mean square residual; BIC: Bayesian Information Criterion. Values in brackets show a confidence interval for RMSEA.

Model fit indices for the one-factor solution were as follows: RMSEA = 0.203 (95% CI; 0.176, 0.235), CFI = 0.835, TLI = 0.617, and SRMR = 0.104. Although absolute fit indices did not meet conventional thresholds, elevated RMSEA values and modest TLI values are likely attributable to the limited sample size and the nature of the construct. The single-factor solution accounted for 32.7% of the variance. Although the single factor explained a relatively modest 32.7% of the variance, this is common and generally acceptable for patient-reported outcome measures assessing complex, subjective behavioural constructs such as listening effort [17,18]. The factor loadings for this solution are presented in Table 3. Because a single-factor solution was retained, cross-loadings were not applicable. Item communalities were generally acceptable, except for Q8 (telephone use), which demonstrated a lower communality (0.276, uniqueness = 0.724).

**Table 3:** Factor loadings for the EEAS questionnaire items.

Question(Q)	Factor 1	Uniqueness
Q1	0.442	0.548
Q2	0.847	0.345
Q3	1.011	0.005
Q4	0.600	0.483
Q5	0.433	0.563
Q6	0.559	0.465
Q7	0.710	0.402
Q8	0.393	0.724
Q9	0.752	0.534
Q10	0.443	0.594

**Note:** The maximum likelihood extraction method was used in combination with oblimin rotation. Factor 1 explained 29.1% of the variance, Factor 2 explained 19.3% variance, and Factor 3 explained 15.0% (total variance explained: 63.4%).

### Convergent validity

Convergent validity was assessed using Pearson’s correlation coefficient to examine the association between the EEAS and VAS, which showed a strong positive correlation (r = 0.611,95% CI:0.473 to 0.722, p < .001), supporting convergent validity. The correlations between the EEAS subscales ranged from r = 0.589 to 0.971. These correlations are presented in Table 4.

**Table 4:** Correlations between EEAS Subscales and Related Measures.

	Mean	SD	EEAS total	EEAS quiet	EEAS noise	EEAS noise3	VAS	Better ear PTA
EEAS Total	4.4	1.9	_					
EEAS*quiet*	3.3	2.2	0.856	_				
EEAS*noise*	4.6	2.1	0.971	0.707	_			
EEAS*noise3*	5.3	2.2	0.871	0.589	0.918	_		
VAS	4.7	2.2	0.611	0.548	0.582	0.556	_	
Better ear PTA	34.6	17.2	0.320	0.299	0.299	0.299	0.449	_

**Note:** All p-values < 0.001. **VAS** = Visual Analogue Scale

### Internal consistency

Internal consistency of the EEAS and its subscales was assessed using Cronbach’s alpha, the total EEAS scale (α = 0.886). The coefficients for the original subscales were EEAS*noise* (α = 0.856), EEAS*noise3* (α = 0.740), and EEAS*quiet* (α = 0.720). Furthermore, all items demonstrated acceptable corrected item-total correlations (>0.30), further supporting the internal consistency of the scale.The results are presented in Table 5.

**Table 5:** Internal consistency measures (Cronbach’s alpha) for EEAS scores.

Subscale domains	Cronbach’s α
**Total EEAS**	0.886
**EEAS*quiet***	0.720
**EEAS*noise***	0.856
**EEAS*noise3***	0.740

**Note:** All Cronbach’s alpha values exceed the conventional threshold of 0.70 for acceptable internal consistency. EEAS*quiet* = items Q1, Q3, Q8; EEAS*noise3* = items Q2, Q4, Q9; EEAS*noise* = items Q2, Q4, Q5, Q6, Q7, Q9, Q10.

### Test-retest reliability

A subset of 20 participants was retested on the EEAS after a two-week interval. The intraclass correlation coefficient (ICC) demonstrated excellent test-retest reliability (ICC = 0.91, 95% CI: 0.82–0.96), as shown in Table 6.

**Table 6:** Test-retest reliability of the EEAS total score.

Measure	n	Test 1	Test 2	ICC	95%CI	SEM
		Mean (SD)	Mean (SD)			
**EEAS Total Score**	20	41.8 (28.4)	40.4 (28.1)	0.91	0.82-0.96	8.41

**Note:** ICC = Intraclass Correlation Coefficient (two-way random effects, single measures); CI = Confidence Interval; SEM = Standard Error of Measurement.

## Rasch analysis

### Item difficulty and item fit statistics

Item difficulty and fit statistics were computed using IRT analysis. Item difficulty is expressed in ‘logits’, which serve as the standard unit of measurement in Rasch modelling, representing the natural-log odds that a participant endorses a higher category. Table 7 presents item difficulties ranging from −0.14 logits for the easiest item (Q1: “Effort to hear in quiet”) to 1.01 logits for the hardest item (Q4: “Concentrate in noise”). This indicates a clear hierarchy of situational listening effort.

**Table 7:** Rasch Item Difficulty and Fit Statistics.

Rank (Difficulty)	Item	Difficulty (Logits)	Infit MNSQ	Outfit MNSQ	Fit Status
1 (Easiest)	Q1	−0.1428	1.27	**1.43**	**Borderline**
2	Q3	−0.0456	1.12	1.23	Acceptable
3	Q8	0.0157	**1.92**	**1.83**	**Misfit**
4	Q6	0.3233	**1.34**	**1.46**	**Borderline**
5	Q10	0.4202	1.18	1.22	Acceptable
6	Q7	0.7438	0.88	1.00	Acceptable
7	Q5	0.8205	0.87	0.98	Acceptable
8	Q2	0.8378	0.91	0.98	Acceptable
9	Q9	0.8761	1.05	1.11	Acceptable
10	Q4	1.0141	**0.64**	**0.69**	**Slight overfit**

**Note:** Item difficulty estimated using a regression-based IRT (Item Response Theory) approach (statsmodels). Fit status is based on MNSQ (Mean-Square) values, with a productive range of 0.70–1.30. Values in **bold** are outside this range.

Item fit was assessed using mean-square (MNSQ) infit and outfit statistics. In Rasch analysis, an ideal fit value is 1.0, indicating that the observed variance perfectly matches the model’s expectations. Values between 0.70 and 1.30 are considered acceptable for measurement (Linacre, 2002). Most items demonstrated acceptable fit. However, three items showed some degree of misfit: Q6 (Outfit MNSQ = 1.46) and Q1 (Outfit MNSQ = 1.43) were borderline, while Q8 (“Listening on telephone in quiet”) showed significant misfit (Infit MNSQ = 1.92; Outfit MNSQ = 1.83). The misfit of Q8 suggests it may measure a slightly different construct or behave unpredictably, a finding that is consistent with its unique behaviour in the earlier factor analysis. Exploratory deletion of Q8 was conducted; however, its removal did not yield a significant improvement in overall person reliability or global model fit. Given the clinical relevance of telephone communication, the item was retained.

The dimensionality of the EEAS-K was further examined using principal component analysis (PCA) of Rasch residuals. The first residual eigenvalue was 1.90. Values below 2.0 are generally regarded as acceptable because they indicate that any secondary dimension has the strength of less than two items, which is statistically indistinguishable from random noise (Linacre, 1998). This indicates that after accounting for the primary listening effort dimension, no meaningful secondary dimension remains in the residuals, supporting the essential one-dimensionality of the EEAS-K in this population.

### Person-item targeting (Wright Map)

**Fig 3:**
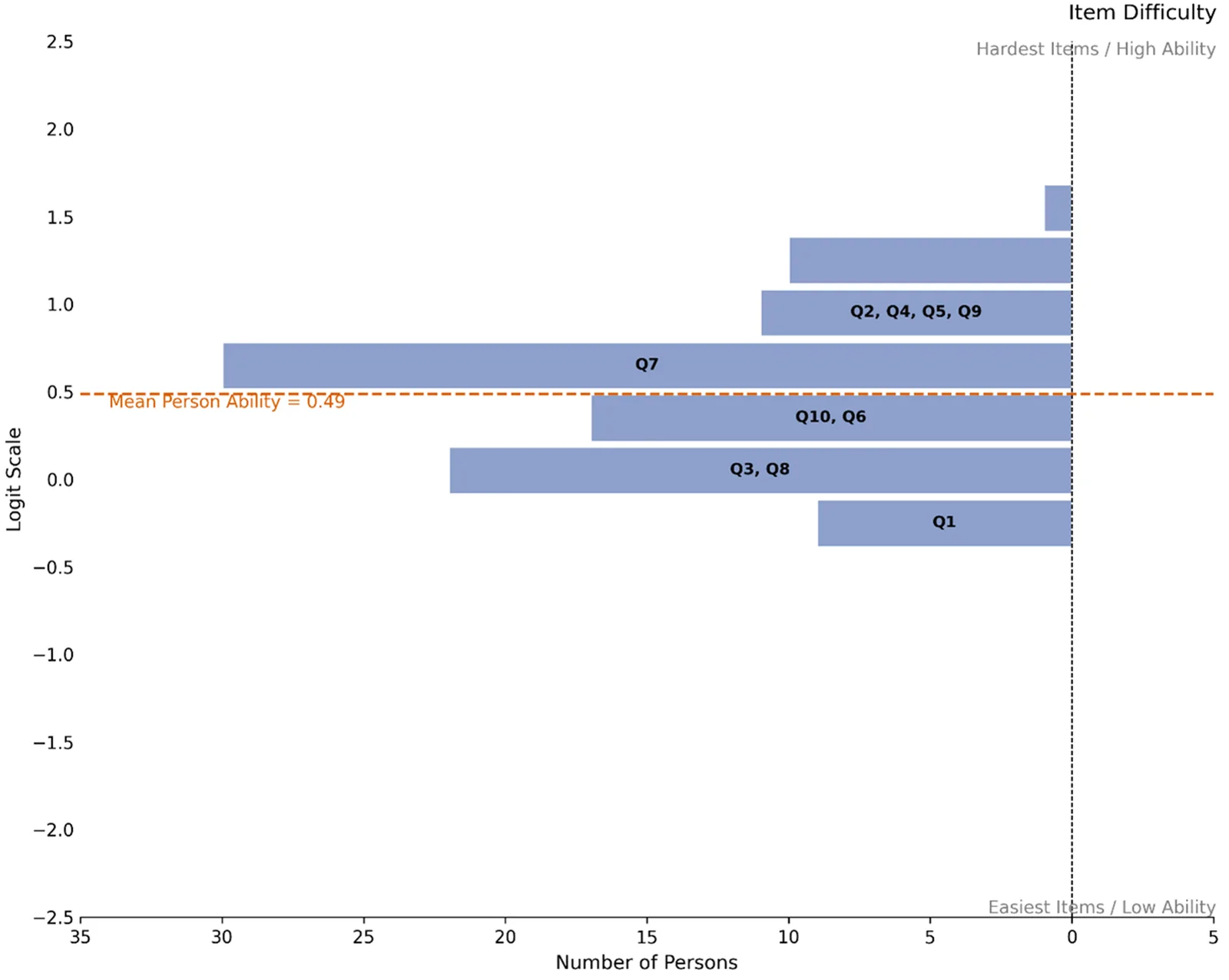
Wright Map for EEAS items. The left side displays the distribution of person abilities (higher values indicate greater perceived listening effort), while the right side displays item difficulties (higher values indicate situations requiring more effort). The dashed horizontal line represents the mean person ability.

Person-item targeting was assessed using a Wright map, which plots the distributions of person abilities and item difficulties on the same logit scale (Fig 3). The mean person ability was 0.49 logits, indicating that, on average, the participants’ level of perceived listening effort was slightly higher than the mean item difficulty of the questionnaire items (centred at 0.00 logits by convention). The Wright map shows the person’s abilities ranged from approximately −0.24 to +1.43 logits, while item difficulties clustered in a narrow range between −0.14 and +1.01 logits. The items are shown clustered within the person distribution bars, suggesting adequate targeting for the clinical sample.

### Differential Item Functioning (DIF)

DIF analysis was conducted to assess item bias across gender, age, and hearing loss severity (better-ear PTA split at 30 dB). As shown in Table 8, the analysis revealed that the scale functions consistently across most subgroups. No significant DIF was found for hearing loss severity on any item. However, significant DIF was observed for Q7 by gender (B = 1.25, p = .003), indicating that females reported greater effort on this item than males of the same ability. Additionally, significant DIF by age was found for Q7 (B = −0.81, p = 0.050) and Q8 (B = 1.69, p = 0.001), suggesting that younger participants reported greater effort on Q7, while older participants reported greater effort on Q8. These differences may reflect variations in daily lifestyle demands, occupational cognitive load, and familiarity with telephone use across different demographic groups. Consequently, scores for Q7 and Q8 should be interpreted with caution across demographic groups.

**Table 8:** Summary of Significant DIF Findings.

Item	Significant DIF	Group Reporting More Effort	p-value	Regression Coefficient (B)
Q7	Gender	Female	0.003	+1.25
	Age	Younger (≤ 53 yrs)	0.050	−0.81
Q8	Age	Older (>53 yrs)	0.001	+1.69

**Note:** No significant DIF (Differential Item Functioning) was found for any other items, p < 0.05. The Regression Coefficient (B) represents the magnitude of the DIF contrast.

## Discussion

The present study involves the cross-cultural adaptation and psychometric validation of the Extended Effort Assessment Scale (EEAS) for the Kannada-speaking population. The Content Validity Index (CVI) for the cultural adaptation of EEAS-K was excellent, indicating that the translated items are highly appropriate for assessing listening effort. This provides a strong foundation for subsequent psychometric validation.

Construct validity was examined using both exploratory factor analysis (EFA) and Rasch/IRT analysis, strongly supporting a unidimensional structure for the EEAS-K. While the original English scale was proposed to be two-dimensional (separating quiet and noisy listening conditions) [9], our findings align with other adapted versions that demonstrated a largely unidimensional structure, where the total score adequately captures the overall construct [10]. Categorising scores by quiet and noise conditions may still be useful as a supplementary guide for clinical interpretation, but the statistical variance is best explained by a single factor. The main difference in the factor structure is likely due to differences in the populations. The current study included a younger demographic (mean age of 48.6 years) with low hearing aid use, whereas the original study included a predominantly older population (median age of 74 years) of experienced hearing aid users. This divergence may reflect differences in the populations studied, though further research is needed to confirm the specific impact of hearing-aid experience on factor structure. In older hearing aid users, the quiet environments are relatively manageable, and noisy environments are considerably more demanding, creating sufficient experiential variance for the two contexts to separate psychometrically. The present sample may perceive listening effort in quiet and in noise as variations of the same underlying challenge rather than qualitatively distinct constructs. Also, there could be an additional burden of central auditory changes with age, which could significantly influence hearing performance in noisy situations in older adults [1]. Taken together, the single general ‘listening effort’ factor better represents the construct in the current population.

Among the individual items, it is noteworthy that Q3, which concerns the effort required to concentrate when listening to someone in a quiet environment, showed a Heywood case in the EFA. The factor loading exceeds unity, and the communality approaches its ceiling. This suggests near-perfect overlap with Q1, which addresses conversational effort in quiet. For younger adults with mild to moderate hearing loss, concentrating during a quiet conversation and having an effective conversation might represent the same cognitive experience. This leaves little room for the items to contribute independently. Alternative 2,3 and 4-factor solutions were explored to resolve this estimation issue; however, these models resulted in uninterpretable factors or single-item factors. Therefore, the 1-factor solution was retained, and the Heywood case was interpreted as the reflection of extreme item redundancy rather than a strictly mathematical artifact. This type of item-level finding, where items that are conceptually distinct in populations with severe hearing loss collapse into a single experience in milder populations, has been documented in cross-population adaptation work [10].

Similarly, Q8, which addresses effort when listening on the telephone in a quiet environment, showed consistent misfit across both EFA and Rasch analysis. This misfit is clinically interpretable rather than problematic, as telephone communication differs from other listening situations due to the absence of visual cues and potential acoustic degradation. Additional factors contributing to this misfit may include the reduction of speech information due to limited telephone bandwidth, varying levels of familiarity with smartphone communication across age groups, and potential issues with hearing aid compatibility during phone use. In the Indian context, among adults under 60 years, text-based communication and streaming often replace voice calls. This may contribute to heterogeneous interpretations of the item across respondents, resulting in inconsistent responses. This finding is consistent with the original validation, which reported that the telephone item had the highest missing response rate in older populations for various reasons [9]. Item revision to reflect contemporary communication technology is recommended for future use with working-age populations.

Rasch/IRT analysis provided further insight into the measurement properties of the EEAS-K. Most items demonstrated acceptable infit and outfit mean square values within the recommended range. The Wright Map demonstrated adequate person-item targeting for this sample, in which items are made marginally easier than the average person’s ability. However, the observed person mean of 0.49 logits indicates that the items were slightly easy for this sample. From a measurement perspective, this indicates that while the scale is highly effective for the general clinical population, it may offer slightly less precision at the extreme upper end of the listening effort spectrum. Nevertheless, this minor ceiling effect does not detract from its overall clinical utility. Differential item functioning (DIF) analysis revealed no significant bias for degree of hearing loss, indicating that the scale functions fairly across the range of hearing impairment levels encountered in this sample, an important finding for clinical use. However, gender-based DIF was observed for Q7, with females reporting greater effort, and age-based DIF was observed for Q7 and Q8, suggesting that younger adults may experience greater effort in group conversations, while older adults report more difficulty with telephone communication. These differences likely reflect variations in daily lifestyle demands and occupational cognitive load [19]. Younger adults and females in this demographic may frequently navigate complex, multi-talker occupational or social environments, thereby experiencing higher subjective effort. Conversely, older adults may struggle more with the degraded acoustic signals of telephone use, whereas younger adults might rely more heavily on text-based communication [1]. These findings provide clinically relevant insights into how listening effort varies across demographic groups and should be considered when interpreting results. While statistically significant, the magnitude of the DIF (as indicated by the regression coefficients) was relatively modest. Therefore, while clinicians should be aware of these demographic nuances, they may not be large enough to necessitate formal score adjustments in routine practice.

The EEAS-Kannada also demonstrated excellent internal consistency, with a Cronbach’s alpha of 0.886, indicating that the items collectively measure a coherent construct. Subscale reliability ranged from acceptable to good (α = 0.720–0.856), further supporting the scale’s reliability. Convergent validity was established through a strong positive correlation with a Visual Analogue Scale for listening effort (r = 0.611, p < .001), confirming that the EEAS-K effectively captures subjective listening effort. Correlations with pure-tone average (PTA) were statistically significant but modest. This is consistent with previous literature, reinforcing the notion that audiometric thresholds alone do not fully reflect the subjective experience of listening effort [20]. Two patients with identical audiograms may experience vastly different levels of daily fatigue and effort. This highlights the critical clinical need for validated self-report tools like the EEAS-K to supplement standard pure-tone audiometry, providing a more holistic picture of the patient’s auditory disability. Finally, the scale demonstrated excellent test–retest reliability (ICC = 0.91), indicating stable and reproducible measurements over time.

A direct evaluation of the EEAS-K alongside the original EEAS demonstrates that it has comparable psychometric properties [9]. In terms of reliability, the EEAS-K exhibited excellent internal consistency (α = 0.886), which closely aligns with the total scale reliability of the original French EEAS (α = 0.92). Regarding item behaviour, the telephone item (Q8) proved to be the most challenging across both populations; it exhibited misfit in our Rasch analysis and similarly showed the highest missing response rate in the original EEAS validation, underscoring the complexity of technology-mediated communication. Finally, while the original EEAS reported a two-factor structure in an older, predominantly aided population, the EEAS-K functions as a unidimensional tool, predominantly in unaided demographics. Overall, the EEAS-K performs similarly to the original instrument across major psychometric indices.

In routine audiology practices, the EEAS-K can serve multiple purposes. It can be used to identify patients experiencing listening effort despite having acceptable pure-tone audiograms, thereby validating their subjective struggles. Furthermore, it can serve as a valuable counselling tool to set realistic expectations, and it has the potential to monitor subjective benefit pre- and post- amplification during the hearing aid fitting process. The excellent test-retest reliability and internal consistency mean that clinicians can confidently use the EEAS-K to establish a stable baseline of a patient’s listening effort.

### Future implication and limitation

The findings of the current study should be interpreted in light of certain methodological constraints. Participants were recruited consecutively from a single tertiary care centre, which may introduce selection bias despite the hospital’s wide catchment area. Additionally, the sample was predominantly non-hearing-aid users (92%). Hearing aid amplification alters the signal-to-noise ratio and subsequent cognitive load, which could fundamentally change how effort is perceived and alter the psychometric behaviour of specific items. Therefore, the generalisation of these findings to experienced hearing aid users remains to be fully determined. The current study restricted inclusion to adults aged 18–59 years who had not undergone formal cognitive screening. Therefore, the applicability of the EEAS-K among older adults needs to be established in future research. Further, while the sample size of 100 participants satisfies traditional subject-to-item ratio heuristics for exploratory factor analysis, it is relatively modest for complex psychometric modelling, which may affect parameter stability. Modern psychometric standards increasingly recommend sample size justifications based on communalities or simulation studies, which should be considered in future validations to ensure parameter stability. The current validation did not include post-intervention responsiveness testing, an important metric that future research should evaluate to confirm the scale’s sensitivity to clinical interventions.

## Conclusion

The EEAS-Kannada (EEAS-K) is a reliable and valid tool for assessing listening effort in Kannada-speaking adults with hearing loss. It demonstrated satisfactory psychometric properties, and the factor structure offers important clinical insights that can contribute to more patient-centred and effective hearing healthcare. The scale shows promise for clinical and research application; however, given the single-centre design, further validation in experienced hearing-aid users and older adult populations will strengthen its generalizability.

## Supporting information

S1 Data Raw dataset. This file contains two sheets. Sheet 1 includes anonymized participant responses (Q1 – Q10) and demographic data used for Exploratory Factor Analysis and Rasch Analysis and includes the total EEAS-K scores from the subset of participants used for the test-retest reliability analysis (Test 1 and Test 2).
(CSV)

S2 File Rasch analysis code. Python script used to calculate item difficulty, fit statistics, person ability, and generate the Wright Map.
(TXT)

S3 File EEAS-K Questionnaire. The final translated 10-item Extended Effort Assessment Scale in Kannada.
(DOCX)

S4 File EEAS Questionnaire (English). The original English version of the Extended Effort Assessment Scale used as the basis for translation.
(DOCX)
