# Supplementary material for: Cross-cultural adaptation and psychometric validation of the Kannada version of the Extended Effort Assessment Scale (EEAS-K) in adults with hearing loss
Source: PLoS One. 2026 Sep 23;21(9):e0358439. doi: 10.1371/journal.pone.0358439 (PMC13600487; doi:10.1371/journal.pone.0358439)
Supplement: Supplementary file 1 [file pone.0358439.s003.docx]

**S3 File. Kannada version of the Extended Effort Assessment Scale (EEAS-K)**

**Note to Editorial Staff and Readers:** This document contains the final translated Kannada-language version of the Extended Effort Assessment Scale (EEAS-K), including the 0-10 rating scale, which is the subject of psychometric validation in this study. It is provided in its original Kannada script for clinical and research use by native speakers. The original English version of this scale is provided separately in S4 File.

**ವಿಸ್ತಾರಿತ ಪ್ರಯತ್ನ ಮೌಲ್ಯಮಾಪನ ಮಾಪಕ**

1) ನೀವು ನಿಶ್ಶಬ್ಧ ವಾತಾವರಣದಲ್ಲಿ ಇರುವಾಗ ಬೇರೆಯವರೊಂದಿಗೆ ಸಂಭಾಷಣೆ ನಡೆಸುವಾಗ ಮಾತುಗಳನ್ನು ಅರ್ಥೈಸಿಕೊಳ್ಳಲು ತುಂಬಾ ಪ್ರಯತ್ನಿಸಬೇಕಾ?

ದಯವಿಟ್ಟು ನಿಮ್ಮ ಪ್ರಯತ್ನದ ಮಟ್ಟವನ್ನು ಕೆಳಗಿನ ಶ್ರೇಣಿಯಲ್ಲಿ ಆಯ್ಕೆಮಾಡಿ:

(0 = ಪ್ರಯತ್ನ ಬೇಕಾಗಿಲ್ಲ, 10 = ಅತಿಯಾದ ಪ್ರಯತ್ನ)

☐ 0 ☐ 1 ☐ 2 ☐ 3 ☐ 4 ☐ 5 ☐ 6 ☐ 7 ☐ 8 ☐ 9 ☐ 10

☐ ಅನ್ವಯಿಸುವುದಿಲ್ಲ

2) ನೀವು ಗದ್ದಲದ ವಾತಾವರಣದಲ್ಲಿ ಇರುವಾಗ ಬೇರೆಯವರೊಂದಿಗೆ ಸಂಭಾಷಣೆ ನಡೆಸುವಾಗ ಮಾತುಗಳನ್ನು ಅರ್ಥೈಸಿಕೊಳ್ಳಲು ತುಂಬಾ ಪ್ರಯತ್ನಿಸಬೇಕಾ?

ದಯವಿಟ್ಟು ನಿಮ್ಮ ಪ್ರಯತ್ನದ ಮಟ್ಟವನ್ನು ಕೆಳಗಿನ ಶ್ರೇಣಿಯಲ್ಲಿ ಆಯ್ಕೆಮಾಡಿ:

(0 = ಪ್ರಯತ್ನ ಬೇಕಾಗಿಲ್ಲ, 10 = ಅತಿಯಾದ ಪ್ರಯತ್ನ)

☐ 0 ☐ 1 ☐ 2 ☐ 3 ☐ 4 ☐ 5 ☐ 6 ☐ 7 ☐ 8 ☐ 9 ☐ 10

☐ ಅನ್ವಯಿಸುವುದಿಲ್ಲ

3) ನೀವು ನಿಶ್ಶಬ್ಧ ವಾತಾವರಣದಲ್ಲಿ ಇರುವಾಗ ಬೇರೆಯವರು ಆಡಿದ ಮಾತನ್ನು ಕೇಳಿಸಿಕೊಳ್ಳಲು ನೀವು ಎಷ್ಟು ಗಮನಕೊಡಬೇಕಾಗುತ್ತದೆ?

ದಯವಿಟ್ಟು ನಿಮ್ಮ ಪ್ರಯತ್ನದ ಮಟ್ಟವನ್ನು ಕೆಳಗಿನ ಶ್ರೇಣಿಯಲ್ಲಿ ಆಯ್ಕೆಮಾಡಿ:

(0 = ಪ್ರಯತ್ನ ಬೇಕಾಗಿಲ್ಲ, 10 = ಅತಿಯಾದ ಪ್ರಯತ್ನ)

☐ 0 ☐ 1 ☐ 2 ☐ 3 ☐ 4 ☐ 5 ☐ 6 ☐ 7 ☐ 8 ☐ 9 ☐ 10

☐ ಅನ್ವಯಿಸುವುದಿಲ್ಲ

4) ನೀವು ಗದ್ದಲದ ವಾತಾವರಣದಲ್ಲಿ ಇರುವಾಗ ಬೇರೆಯವರು ಆಡಿದ ಮಾತನ್ನು ಕೇಳಿಸಿಕೊಳ್ಳಲು ನೀವು ಎಷ್ಟು ಗಮನಕೊಡಬೇಕಾಗುತ್ತದೆ?

ದಯವಿಟ್ಟು ನಿಮ್ಮ ಪ್ರಯತ್ನದ ಮಟ್ಟವನ್ನು ಕೆಳಗಿನ ಶ್ರೇಣಿಯಲ್ಲಿ ಆಯ್ಕೆಮಾಡಿ:

(0 = ಪ್ರಯತ್ನ ಬೇಕಾಗಿಲ್ಲ, 10 = ಅತಿಯಾದ ಪ್ರಯತ್ನ)

☐ 0 ☐ 1 ☐ 2 ☐ 3 ☐ 4 ☐ 5 ☐ 6 ☐ 7 ☐ 8 ☐ 9 ☐ 10

☐ ಅನ್ವಯಿಸುವುದಿಲ್ಲ

5) ಏನನ್ನಾದರೂ ಕೇಳಿಸಿಕೊಳ್ಳುವಾಗ ಇತರ ಶಬ್ದಗಳನ್ನು ನೀವು ಎಷ್ಟು ಸುಲಭವಾಗಿ ನಿರ್ಲಕ್ಷಿಸಬಹುದು ?

ದಯವಿಟ್ಟು ನಿಮ್ಮ ಪ್ರಯತ್ನದ ಮಟ್ಟವನ್ನು ಕೆಳಗಿನ ಶ್ರೇಣಿಯಲ್ಲಿ ಆಯ್ಕೆಮಾಡಿ:

(0 = ಪ್ರಯತ್ನ ಬೇಕಾಗಿಲ್ಲ, 10 = ಅತಿಯಾದ ಪ್ರಯತ್ನ)

☐ 0 ☐ 1 ☐ 2 ☐ 3 ☐ 4 ☐ 5 ☐ 6 ☐ 7 ☐ 8 ☐ 9 ☐ 10

☐ ಅನ್ವಯಿಸುವುದಿಲ್ಲ

6) ನಿಮಗೆ ತರಗತಿಯಲ್ಲಿ , ಸಭೆಗಳಲ್ಲಿ ಅಥವಾ ಉಪನ್ಯಾಸವನ್ನು ಅನುಸರಿಸಲು ತುಂಬಾ ಪ್ರಯತ್ನ ಮಾಡಬೇಕಾಗುತ್ತದೆಯೇ ?

ದಯವಿಟ್ಟು ನಿಮ್ಮ ಪ್ರಯತ್ನದ ಮಟ್ಟವನ್ನು ಕೆಳಗಿನ ಶ್ರೇಣಿಯಲ್ಲಿ ಆಯ್ಕೆಮಾಡಿ:

(0 = ಪ್ರಯತ್ನ ಬೇಕಾಗಿಲ್ಲ, 10 = ಅತಿಯಾದ ಪ್ರಯತ್ನ)

☐ 0 ☐ 1 ☐ 2 ☐ 3 ☐ 4 ☐ 5 ☐ 6 ☐ 7 ☐ 8 ☐ 9 ☐ 10

☐ ಅನ್ವಯಿಸುವುದಿಲ್ಲ

7) ತುಂಬಾ ಗದ್ದಲವಿರುವ ವಾತಾವರಣದಲ್ಲಿ ಇತರರೊಂದಿಗೆ ಸಂಭಾಷಣೆಯನ್ನು ಅನುಸರಿಸಲು ತುಂಬಾ ಪ್ರಯತ್ನ ಮಾಡಬೇಕಾಗುತ್ತದೆಯೇ? ( ಹೋಟೆಲಿನಲ್ಲಿ , ಕುಟುಂಬದವರು ಒಟ್ಟು ಸೇರಿದಾಗ)

ದಯವಿಟ್ಟು ನಿಮ್ಮ ಪ್ರಯತ್ನದ ಮಟ್ಟವನ್ನು ಕೆಳಗಿನ ಶ್ರೇಣಿಯಲ್ಲಿ ಆಯ್ಕೆಮಾಡಿ:

(0 = ಪ್ರಯತ್ನ ಬೇಕಾಗಿಲ್ಲ, 10 = ಅತಿಯಾದ ಪ್ರಯತ್ನ)

☐ 0 ☐ 1 ☐ 2 ☐ 3 ☐ 4 ☐ 5 ☐ 6 ☐ 7 ☐ 8 ☐ 9 ☐ 10

☐ ಅನ್ವಯಿಸುವುದಿಲ್ಲ

8) ನೀವು ನಿಶ್ಶಬ್ಧ ವಾತಾವರಣದಲ್ಲಿ ಇರುವಾಗ ನಿಮಗೆ ದೂರವಾಣಿಯಲ್ಲಿ (ಟೆಲೆಫೋನಿನಲ್ಲಿ) ಕೇಳಿಸಿಕೊಳ್ಳಲು ತುಂಬಾ ಪ್ರಯತ್ನ ಮಾಡಬೇಕಾಗುತ್ತದೆಯೇ ?

ದಯವಿಟ್ಟು ನಿಮ್ಮ ಪ್ರಯತ್ನದ ಮಟ್ಟವನ್ನು ಕೆಳಗಿನ ಶ್ರೇಣಿಯಲ್ಲಿ ಆಯ್ಕೆಮಾಡಿ:

(0 = ಪ್ರಯತ್ನ ಬೇಕಾಗಿಲ್ಲ, 10 = ಅತಿಯಾದ ಪ್ರಯತ್ನ)

☐ 0 ☐ 1 ☐ 2 ☐ 3 ☐ 4 ☐ 5 ☐ 6 ☐ 7 ☐ 8 ☐ 9 ☐ 10

☐ ಅನ್ವಯಿಸುವುದಿಲ್ಲ

9) ನೀವು ಗದ್ದಲದ ವಾತಾವರಣದಲ್ಲಿ ಇರುವಾಗ ನಿಮಗೆ ದೂರವಾಣಿಯಲ್ಲಿ (ಟೆಲೆಫೋನಿನಲ್ಲಿ) ಕೇಳಿಸಿಕೊಳ್ಳಲು ತುಂಬಾ ಪ್ರಯತ್ನ ಮಾಡಬೇಕಾಗುತ್ತದೆಯೇ ?

ದಯವಿಟ್ಟು ನಿಮ್ಮ ಪ್ರಯತ್ನದ ಮಟ್ಟವನ್ನು ಕೆಳಗಿನ ಶ್ರೇಣಿಯಲ್ಲಿ ಆಯ್ಕೆಮಾಡಿ:

(0 = ಪ್ರಯತ್ನ ಬೇಕಾಗಿಲ್ಲ, 10 = ಅತಿಯಾದ ಪ್ರಯತ್ನ)

☐ 0 ☐ 1 ☐ 2 ☐ 3 ☐ 4 ☐ 5 ☐ 6 ☐ 7 ☐ 8 ☐ 9 ☐ 10

☐ ಅನ್ವಯಿಸುವುದಿಲ್ಲ

10) ಪ್ರಕಟಿಸಲಾದ ಘೋಷಣೆಯನ್ನು ಕೇಳಿಸಿಕೊಳ್ಳಲು ತುಂಬಾ ಪ್ರಯತ್ನ ಮಾಡಬೇಕಾಗುತ್ತದೆಯೆ ? ( ರೈಲು ನಿಲ್ದಾಣ , ವಿಮಾನ ನಿಲ್ದಾಣ , ಅಂಡರ್ ಗ್ರೌಂಡ್/ ಭೂಗರ್ಭ ನಿಲ್ದಾಣ, ಕ್ರೀಡಾಂಗಣ ಅಥವಾ ಮಾರುಕಟ್ಟೆಯಲ್ಲಿ)

ದಯವಿಟ್ಟು ನಿಮ್ಮ ಪ್ರಯತ್ನದ ಮಟ್ಟವನ್ನು ಕೆಳಗಿನ ಶ್ರೇಣಿಯಲ್ಲಿ ಆಯ್ಕೆಮಾಡಿ:

(0 = ಪ್ರಯತ್ನ ಬೇಕಾಗಿಲ್ಲ, 10 = ಅತಿಯಾದ ಪ್ರಯತ್ನ)

☐ 0 ☐ 1 ☐ 2 ☐ 3 ☐ 4 ☐ 5 ☐ 6 ☐ 7 ☐ 8 ☐ 9 ☐ 10   ☐ ಅನ್ವಯಿಸುವುದಿಲ್ಲ
