# Supplementary material for: Cross-cultural adaptation and psychometric validation of the Kannada version of the Extended Effort Assessment Scale (EEAS-K) in adults with hearing loss
Source: PLoS One. 2026 Sep 23;21(9):e0358439. doi: 10.1371/journal.pone.0358439 (PMC13600487; doi:10.1371/journal.pone.0358439)
Supplement: Supplementary file 2 [file pone.0358439.s004.docx]

**Extended Effort Assessment Scale (EEAS) – Original English Version**

**Items:**

1. Do you have to put in a lot of effort to hear what is being said in conversation with others, in a quiet environment?
2. Do you have to put in a lot of effort to hear what is being said in conversation with others, in a noisy environment?
3. How much do you have to concentrate when listening to someone, in a quiet environment?
4. How much do you have to concentrate when listening to someone, in a noisy environment?
5. How easily can you ignore other sounds when trying to listen to something?
6. Do you have to put in a lot of effort to follow discussion in a class, a meeting, or a lecture?
7. Do you have to put in a lot of effort to follow the conversation in a noisy environment (e.g., in a restaurant, at family gatherings)?
8. Do you have to put in a lot of effort to listen on the telephone, in a quiet environment?
9. Do you have to put in a lot of effort to listen on the telephone, in a noisy environment?
10. Do you have to put in a lot of effort to understand announcements made by loudspeakers, in train stations, airports, underground stations, stadium or a supermarket?

Attribution Note:

These items are reproduced from the original Extended Effort Assessment Scale. Permission to translate and adapt this scale into Kannada was explicitly obtained from the original authors prior to the commencement of this study.

**Original Source:** *Ferschneider M, Moulin A. Listening Effort in Quiet and Noisy Environments in the Daily Life of Adults With Hearing Aids: An Extended Version of the Effort Assessment Scale (EEAS). Trends in Hearing. 2023. DOI: 10.1177/23312165231176320*
